# Image‐based robotics enhance precision and efficiency in lateral UKA: A comparative study of 135 UKAs

**DOI:** 10.1002/ksa.70012

**Published:** 2025-09-09

**Authors:** Clément Favroul, Cécile Batailler, Elsayed Ahmed Abdelatif, Elvire Servien, Sébastien Lustig

**Affiliations:** ^1^ Orthopaedics Surgery and Sports Medicine Department, FIFA Medical Center of Excellence, Croix‐Rousse Hospital, Hospices Civils de Lyon Lyon North University Hospital Lyon France; ^2^ Univ Lyon, Claude Bernard Lyon 1 University, IFSTTAR, LBMC UMR_T9406 Lyon France; ^3^ Department of Orthopaedic Surgery and Traumatology, Faculty of Medecine Minia University Minia Egypt; ^4^ LIBM‐EA 7424, Interuniversity Laboratory of Biology of Mobility Claude Bernard Lyon 1 University Lyon France

**Keywords:** image‐based, imageless, lateral unicompartmental knee arthroplasty, robotic surgery

## Abstract

**Purpose:**

Robotic‐assisted lateral unicompartmental knee arthroplasty (UKA) remains technically demanding due to the complex biomechanics of the lateral compartment. Image‐based (IBRA) and imageless (ILRA) robotic systems have both demonstrated superior accuracy compared to conventional mechanical instrumentation, but have not yet been directly compared in lateral UKA. This study aimed to evaluate their respective accuracy and surgical efficiency.

**Methods:**

This retrospective study included 135 patients who underwent lateral UKA using either IBRA or ILRA systems. Post‐operative radiographic outcomes included hip–knee–ankle (HKA) alignment, posterior tibial slope (PTS) and joint line (JL) restoration. Surgical time was assessed as a secondary outcome. Target zones were HKA 180°–185°, PTS 2°–8° and JL ± 2 mm.

**Results:**

IBRA showed higher rates of HKA inliers (94.9% vs. 78.9%, *p* = 0.011) and JL restoration (76.2% vs. 31.6%, *p* < 0.001) compared to ILRA. PTS accuracy was similar between groups (*p* = 0.30). Operative time was significantly shorter with IBRA (61.6 ± 13.5 vs. 81.9 ± 26.1 min, *p* < 0.001).

**Conclusion:**

IBRA outperformed ILRA in terms of alignment accuracy and surgical duration. These findings support the added value of IBRA systems in lateral UKA.

**Level of Evidence:**

Level IV, retrospective case series study.

**Abbreviations**
BMI: body mass index
HKA: hip–knee–ankle
IBRA: image‐based robotic‐assisted
ILRA: imageless robotic‐assisted
JL: joint line
LTFO: lateral tibiofemoral osteoarthritis
OA: osteoarthitis
PTS: posterior tibial slope
RA: robotic‐assisted
UKA: unicompartmental knee arthroplasty

## INTRODUCTION

Unicompartmental knee arthroplasty (UKA) is a well‐established treatment for isolated knee osteoarthritis, most commonly affecting the medial compartment [23]. In contrast, lateral UKA accounts for only about 10% of all UKA procedures [26, 30]. Its lower adoption is partly due to the greater technical demands of the procedure, which are related to the distinct biomechanics of the lateral compartment—including the physiological laxity in flexion and the screw‐home mechanism [14, 29]. These features make implant positioning more complex and increase the risk of malalignment.

Implant malposition is one of the main causes of UKA failure [3, 11, 13]. In the lateral compartment specifically, malalignment can result in uneven load distribution, joint instability, abnormal kinematics and persistent pain, all of which contribute to early mechanical failure and increased revision rates [7, 8, 9, 25]. Accurate alignment and restoration of the joint line (JL) are therefore critical to ensure implant longevity and optimal functional outcomes.

Robotic‐assisted (RA) surgery has been shown to improve alignment accuracy compared to conventional instrumentation [10, 12, 15, 28, 34], with particular benefit in complex procedures such as lateral UKA [4, 13, 27, 35]. By offering enhanced preoperative planning or real‐time intraoperative mapping, RA systems can help replicate native anatomy and reduce outliers in implant positioning [6, 17].

Over the past few years, RA systems for UKA have evolved into two main categories: image‐based (IBRA) and imageless (ILRA) systems [33]. IBRA utilizes preoperative CT scans to generate a 3D model of the patient's knee. ILRA, on the other hand, relies solely on intraoperative bone mapping to guide the surgery in real time. Both techniques have improved implant accuracy compared to traditional manual methods [10, 12, 15, 28, 34]. To date, no study has directly compared IBRA and ILRA systems in lateral UKA, leaving a gap in the literature regarding their respective accuracy and efficiency in this technically demanding procedure.

The aim of this study was to assess whether two RA systems could achieve accurate post‐operative alignment within a target zone of 180°–185° for valgus knees, while adapting to each patient's anatomical features. The target zone served as a clinical reference, but implant positioning was planned individually, based on the patient's native alignment and contralateral limb when available. We hypothesized that the IBRA system offers superior precision in reproducing the patient's native anatomy and achieving optimal implant positioning compared to the ILRA system. Operative time was evaluated as a secondary outcome.

## MATERIALS AND METHODS

### Patients population

**Figure 1:**
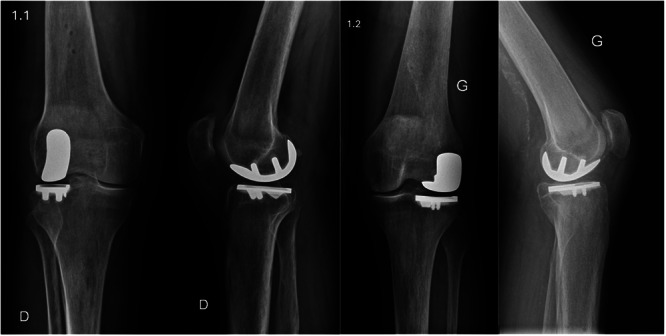
Implant designs: (1.1) Post‐operative anteroposterior and lateral radiographs of a right knee following IBRA‐assisted UKA with the MAKO system. (1.2) Post‐operative anteroposterior and lateral radiographs of a left knee following ILRA‐assisted UKA with the NAVIO system. IBRA, image‐based robotic‐assisted; UKA, unicompartmental knee arthroplasty.

A retrospective, comparative radiographic investigation was carried out on patients who presented with lateral femorotibial osteoarthritis (LTFO), essential or secondary to either post‐traumatic changes or previous meniscectomy, all of whom were treated via lateral UKA. Two different robotic approaches were evaluated: an IBRA system (Restoris MCK, MAKO®, Stryker Corporation) and an ILRA system (NAVIO® Journey 1 UKA, Smith & Nephew) (Figure 1). Patients undergoing ILRA‐UKA between October 2013 and May 2024 were included alongside those who received IBRA‐UKA from April 2022 to June 2024. During this period, a total of 194 lateral UKAs were performed at our institution, of which 135 were carried out using robotic assistance. From 2022 to 2024, lateral UKAs were preferentially performed with the IBRA system, while the ILRA system was used occasionally, depending on system availability. All procedures took place at a single centre and were performed by three experienced surgeons, each conducting more than 30 UKAs per year, following a consistent surgical protocol. Exclusion criteria included the need for concomitant procedures (e.g., ligament reconstruction, bi‐unicompartmental arthroplasty, patellofemoral arthroplasty), coronal deformity over 20° of valgus, inability to bend the knee beyond 90°, flexion contractures exceeding 15°, and clinical instability in the coronal or sagittal plane. Revision UKA cases were also excluded. In contrast, while age and baseline activity level were not considered exclusion criteria, patients with a body mass index (BMI) > 40 kg/m^2^ were excluded. Although recent studies suggest that UKA can be performed safely in obese patients [19, 31], RA procedures in this population remain technically more challenging—particularly in terms of registration accuracy and intraoperative visualization. This exclusion also ensured consistent inclusion criteria across the 10‐year study period. A review of preoperative patient records was conducted to collect information on demographic variables (age, sex and BMI) and details about the aetiology of LTFO (Table 1).

**Table 1:** Demographic characteristics for IBRA and ILRA groups.

Parameters, *N* (%) mean ± SD [min–max]	IBRA group (*N* = 59)	ILRA group (*N* = 76)	*p* **value**
Gender	F: 41 (69.5%)	F: 51 (67.1%)	0.76
	M: 18 (30.5%)	M: 25 (32.9%)	
Age (y‐o)	65.9 ± 12.7 [31–91]	65.1 ± 14.0 [20–93]	0.70
BMI (kg/m^2^)	25.2 ± 4.2 [18–36]	24.7 ± 4.6 [18–39]	0.45
Aetiology	Essential: 25 (42%)	Essential: 32 (42%)	0.75
	Post‐traumatic: 6 (10%)	Post‐traumatic: 11 (14%)	
	Meniscectomy: 27 (46%)	Meniscectomy: 33 (44%)	
	SPONK: 1 (2%)	SPONK: 0 (0%)	
Length of surgery (min)	61.6 ± 13.5 [36–105]	81.9 ± 26.1 [52–189]	<0.001
Pre‐op HKA (°)	184.3 ± 3.7 [175–194]	186.2 ± 5.1 [177–200]	0.01
Post‐op HKA (°)	181.5 ± 2.0 [177–187]	181.6 ± 2.2 [177–189]	0.68
Difference HKA	−2.8 ± 3.4 [−13 to 8]	−4.6 ± 3.4 [−14 to 5]	0.001
Inliers HKA	56/59 (94.9%)	60/76 (78.9%)	0.011
Pre‐op PTS (°)	7.6 ± 3.8 [−1 to 19]	7.4 ± 2.3 [−2 to 16]	0.69
Post‐op PTS (°)	4.2 ± 3.0 [−2 to 12]	3.8 ± 2.4 [0–11]	0.30
Difference PTS (°)	−3.4 ± 3.9 [−13 to 6]	−3.6 ± 3.6 [−12 to 6]	0.31
Inliers PTS (°)	39 (66.1%)	53 (69.7%)	0.66
Post‐op JL (mm)	0.93 ± 1.5 [−4 to 5]	2.5 ± 2.5 [−6 to 8]	<0.001
JL restoration within ±2 mm	45 (76.2%)	25 (31.6%)	<0.001

Abbreviations: BMI, body mass index; F, female; HKA, hip–knee–ankle angle; IBRA, imaged‐based robotic assistance; ILRA, imageless robotic assistance; JL, joint line; M, male; Max, maximum; Min, minimum; Post‐op, post‐operative; Pre‐op, preoperative; PTS, posterior tibial slope; SD, standard deviation; °, degrees.

### Surgery

In the group receiving an IBRA procedure, a preoperative CT scan was obtained, enabling the construction of a 3D model of the lateral femorotibial joint. This model provided an individualized template for implant positioning. Surgeons generally targeted a residual lateral compartment laxity of about 2–4 mm at around 15° and 90° of knee flexion.

For the ILRA cohort, the 3D bone model was established intraoperatively by a bone morphing step. Although the workflows differed—IBRA relying on preoperative imaging versus ILRA generating the model in real time—both systems embraced the same fundamental objectives: accurate sizing of the prosthetic components, respect for native knee kinematics, and maintenance of the screw‐home mechanism.

All procedures employed a minimally invasive lateral approach, with each surgeon following standardized protocols specific to the relevant robotic system. Alignment targets were initially defined within a standardized post‐operative range of 180°–185° for valgus knees, in accordance with commonly accepted radiographic thresholds used to assess coronal alignment accuracy in robotic UKA. However, these benchmarks served primarily as a reference framework rather than an absolute objective. Surgical planning incorporated individual patient‐specific anatomical parameters, including the mechanical axis of the contralateral limb—when not affected by deformity—as well as the overall lower limb alignment and morphology. In cases of lateral compartment arthritis in varus‐aligned knees, the alignment objective was to achieve normocorrection to avoid persistent medial overload. Final intraoperative alignment decisions were based on the reducibility of the deformity and real‐time assessment of soft tissue tension, bony anatomy, and joint kinematics, allowing implant positioning to be adjusted as needed to preserve the patient's native biomechanical profile whenever appropriate. Across all patients, an additional objective was to restore posterior tibial slope (PTS) in a manner consistent with the patient's preoperative anatomy, while prioritizing optimal ligament balancing and joint kinematics. Although a target range of 2°–8° was used as a general guideline—based on prior studies [5, 16] supporting its safety and functional relevance—final PTS was adapted case‐by‐case. JL height was also corrected with a target deviation of no more than ±2 mm.

### Radiographical assessment

**Figure 2:**
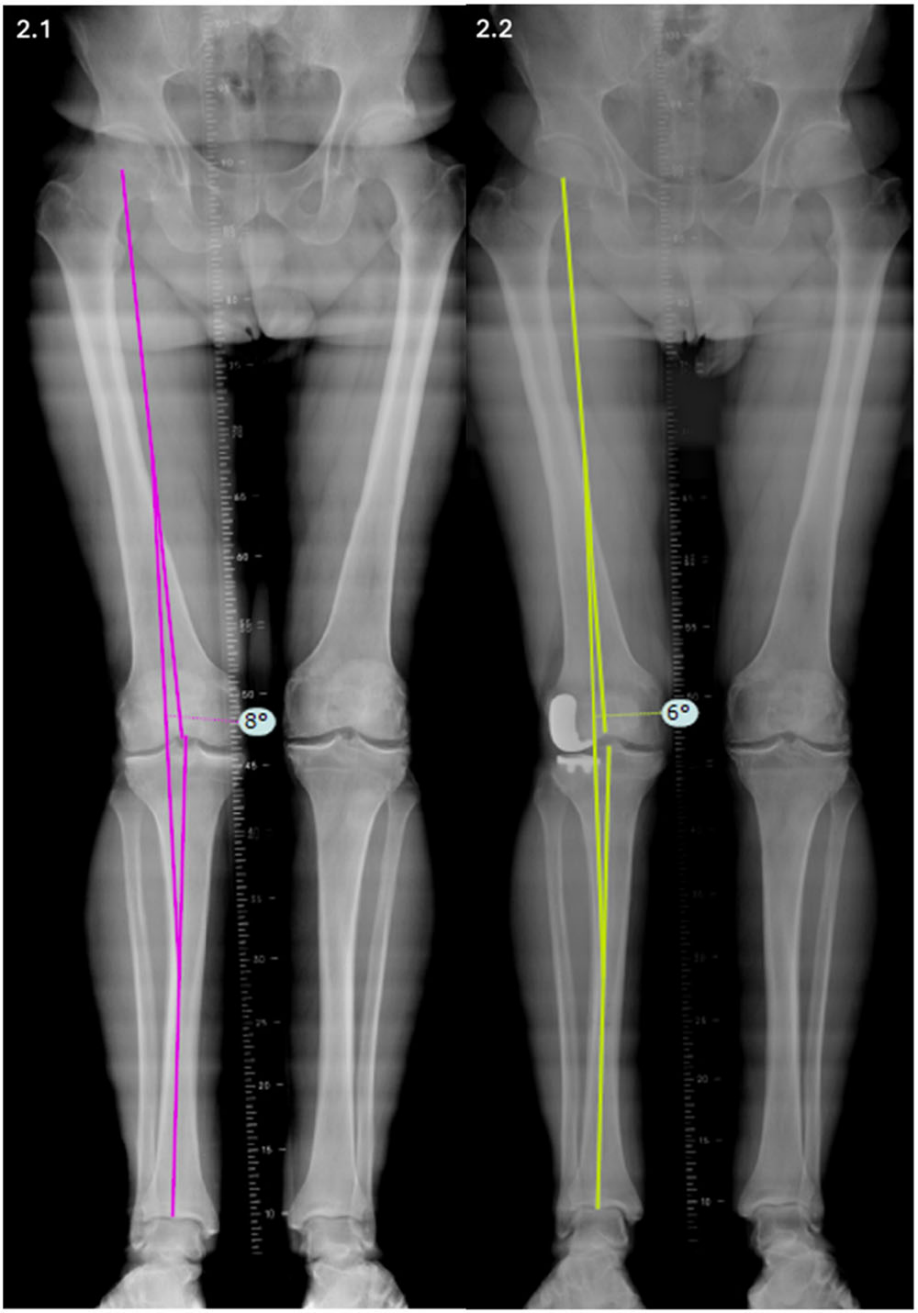
Full‐length radiograph of the lower limbs illustrating the assessment of the hip–knee–ankle angle in (2.1) preoperative and (2.2) post‐operative conditions.

**Figure 3:**
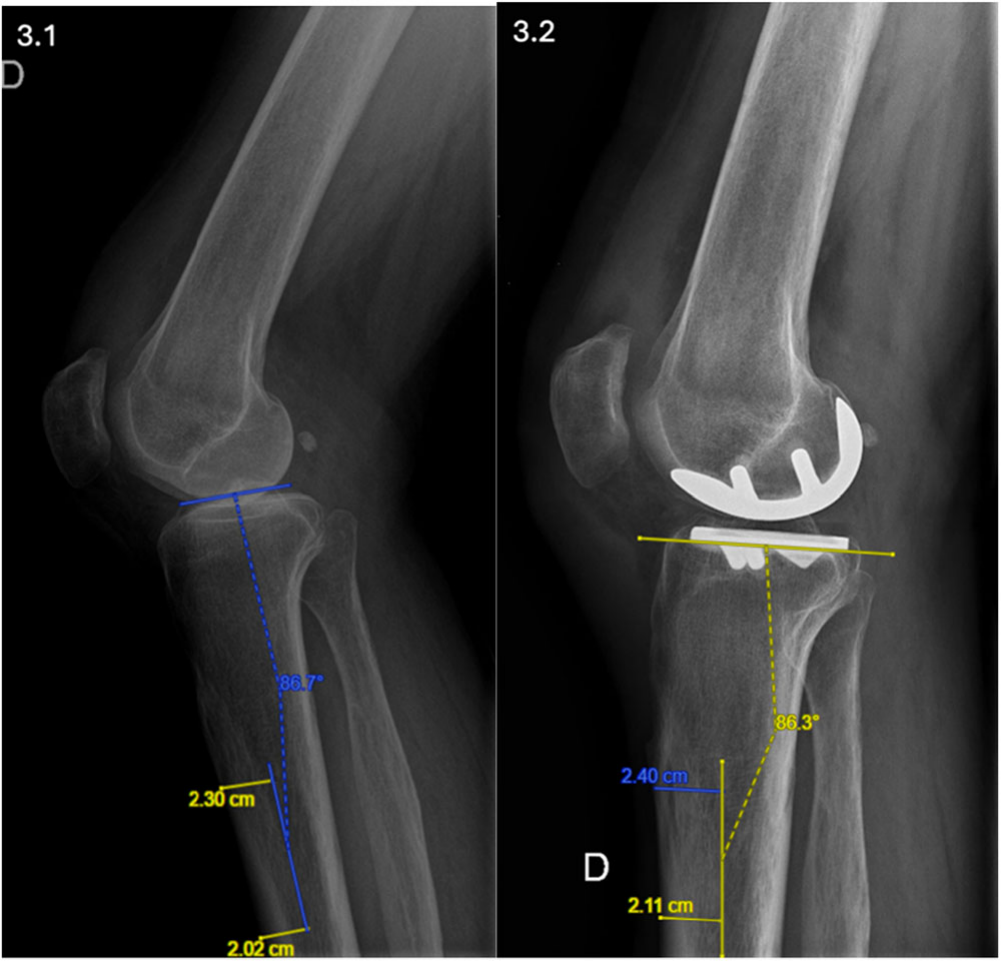
Lateral radiograph of the right knee illustrating the measurement of the lateral tibial plateau slope in (3.1) preoperative and (3.2) post‐operative conditions. The lateral PTS is defined as the angle between the lateral tibial plateau and a line passing through the centre of the tibial diaphysis on the lateral view. PTS, posterior tibial slope.

**Figure 4:**
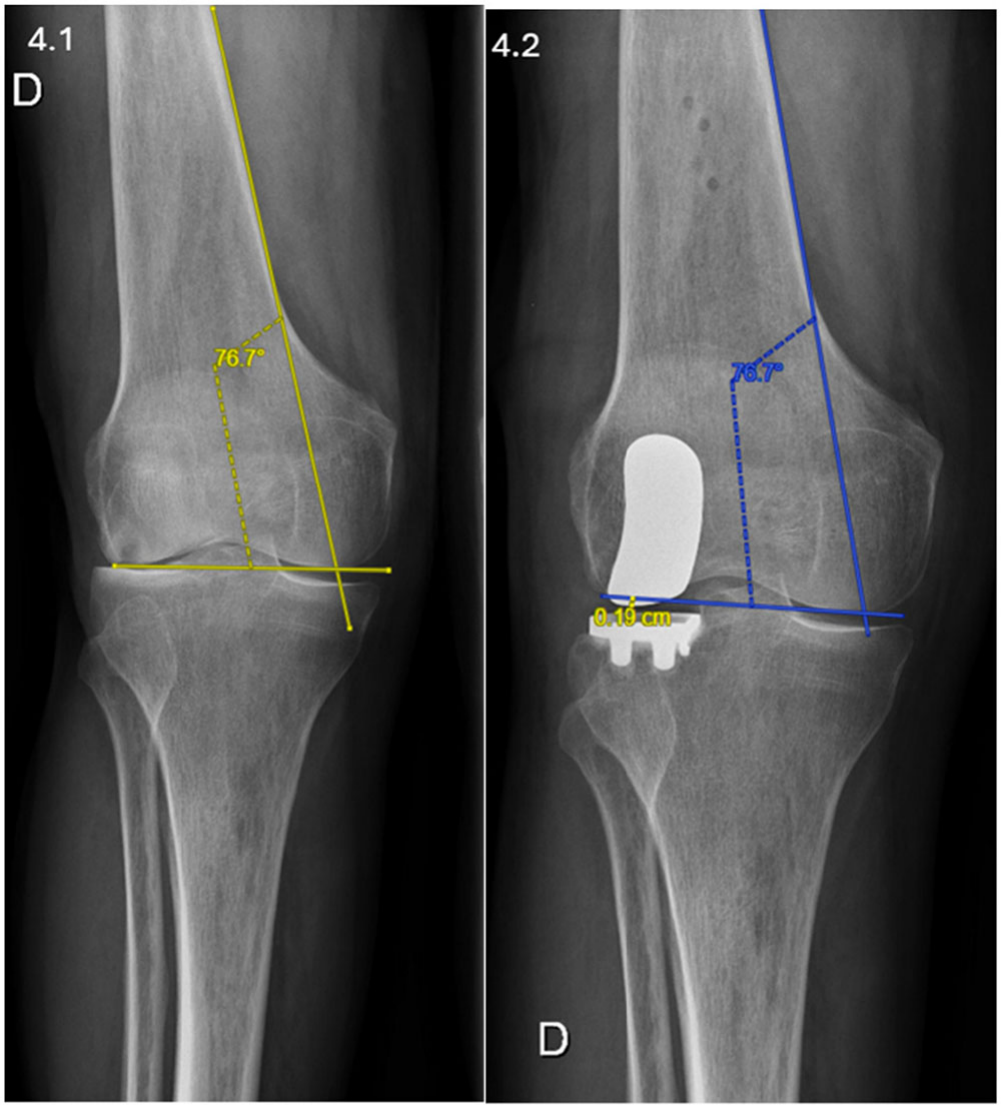
Joint line height measurement using Herry's method. Assessment of joint line height modification post‐operatively, showing a distalization of 1.9 mm. The joint line is initially determined by the angle formed between a line tangential to the medial femoral cortex and another passing through the most distal point of the femoral condyles (4.1). Post‐operatively, this preoperative angle is reapplied to the medial femoral cortex and the distal end of the medial femoral condyle, with the vertical distance between the most distal point of the femoral component and this reference line quantified as the change in joint line height (4.2), following the method validated by Herry et al. [15].

Preoperatively, and then at 3 and 6 months post‐operatively, patients underwent weight‐bearing anteroposterior (AP) and lateral knee radiographs, axial patellofemoral views and full long leg standing radiographs. These radiographs were used to assess implant positioning and alignment before surgery and at the 6‐month follow‐up. Preoperative measurements focused on the HKA angle, acquired from full long leg radiographs (Figure 2), and the PTS in the lateral compartment defined as the angle between the lateral tibial plateau and a line passing through the centre of the tibial diaphysis on the lateral view (Figure 3). Post‐operative assessments included HKA, PTS, tibial component alignment in the coronal plane and restoration of the JL, defined as positive when the JL was lowered and negative when it was raised relative to preoperative levels (Figure 4).

All radiographs underwent evaluation by two independent knee surgeons (CF and AE) using Centricity Universal Viewer Zero Footprint (version 6.0 SP7.0.2—GE Healthcare), which enables angle measurements with an accuracy of 0.1°. Post‐operative results were classified as outliers if HKA fell outside 180–185°, if PTS was below 2° or above 8°, or if the JL height changed by more than ±2 mm.

### Statistical analysis

For all continuous variables, descriptive statistics included mean values, standard deviations and the corresponding minimum and maximum. The normality of the data distribution was evaluated using the Shapiro–Wilk test. When the assumption of normality was met, comparisons were made using a t‐test; otherwise, non‐parametric analyses (Mann–Whitney *U* or Wilcoxon rank‐sum tests) were applied. Categorical variables were summarized as percentages, and differences between groups were assessed with either the chi‐square or Fisher's exact test, depending on expected cell counts. Statistical significance was set at *p* < 0.05.

Intraobserver reliability was excellent, with Pearson correlation coefficients of *ρ* = 0.95 (95% confidence interval [CI]: [0.90–0.98]; *p* < 0.001) for the post‐operative HKA measurement, *ρ* = 0.91 (95% CI: [0.84–0.94]; *p* < 0.001) for JL restoration, and *ρ* = 0.93 (95% CI: [0.90–0.97]; *p* < 0.001) for the post‐operative tibial slope. Interobserver reliability was also high, with *ρ* = 0.86 (95% CI: [0.78–0.90]; *p* < 0.001) for HKA, *ρ* = 0.83 (95% CI: [0.77–0.88]; *p* < 0.001) for JL restoration and *ρ* = 0.90 (95% CI: [0.86–0.94]; *p* < 0.001) for tibial slope.

All statistical analyses were carried out using XLSTAT™ (2021, AddInsoft).

### Ethical approval

All procedures were performed in accordance with the ethical standards of the institutional and/or national research committee, the 1964 Helsinki Declaration and its later amendments, or comparable ethical standards. Data collection and analysis were carried out in accordance with MR004 Reference Methodology from the Commission Nationale de l'Informatique et des Libertés (Ref. 2229975V0) obtained on 6 May 2023. The study was registered and filed on the Health Data Hub website.

## RESULTS

The demographic and clinical characteristics of the patients were comparable between the IBRA and ILRA groups (Table 1). Both groups had similar distributions of gender, age, BMI and aetiology.

The surgical time was significantly shorter for the IBRA group (61.6 ± 13.5 min for IBRA vs. 81.9 ± 26.1 min for ILRA, *p* < 0.001).

**Figure 5:**
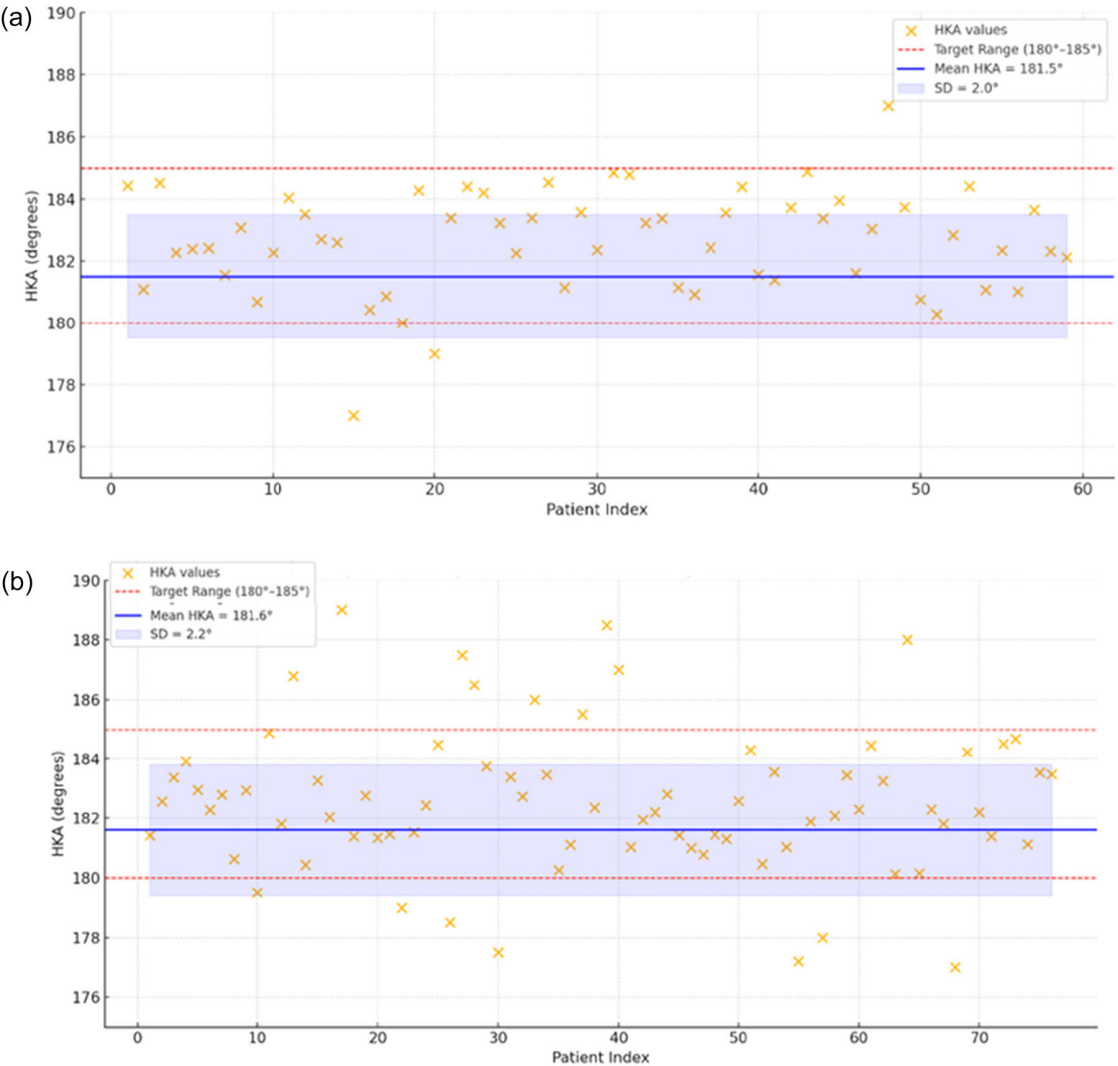
Graphic representation of radiographic results for HKA for IBRA (a) and ILRA (b). HKA, hip–knee–ankle; IBRA, image‐based robotic‐assisted.

**Figure 6:**
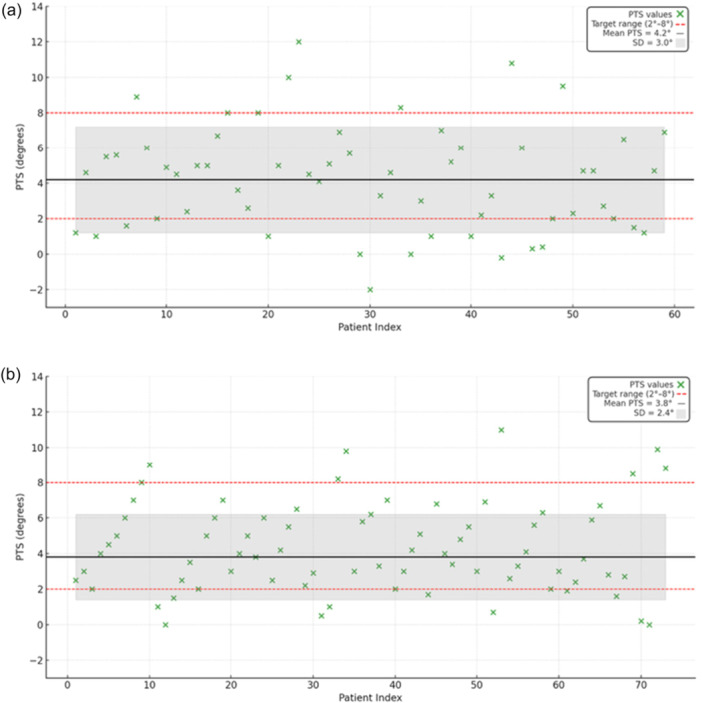
Graphic representation of radiographic results for PTS for IBRA (a) and ILRA (b). IBRA, image‐based robotic‐assisted; PTS, posterior tibial slope.

**Figure 7:**
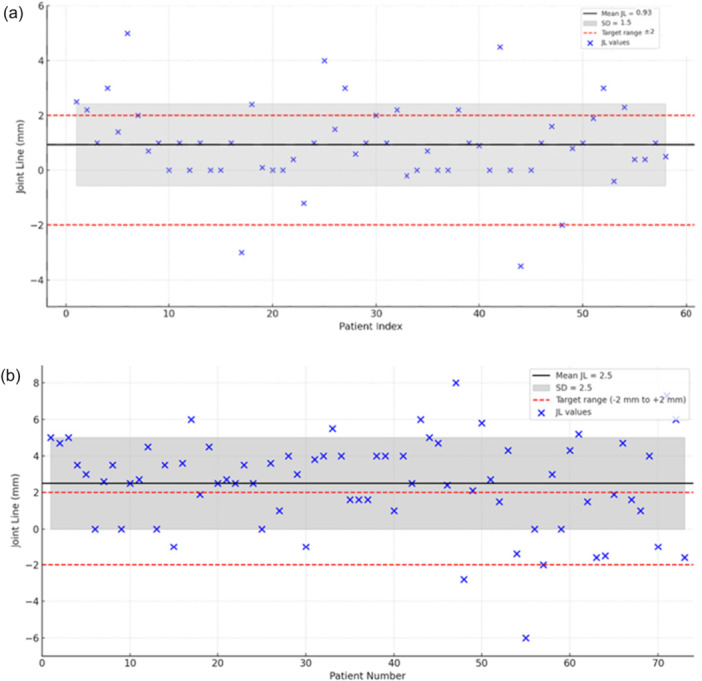
Graphic representation of radiographic results for JL for IBRA (a) and ILRA (b). IBRA, image‐based robotic‐assisted; JL, joint line.

Post‐operative alignment results are summarized in Figures 5, 6, 7 and Table 1.

For the HKA (Figure 5), a significantly greater proportion of patients in the IBRA group achieved post‐operative HKA alignment within the target range compared to the ILRA group (94.9% vs. 78.9%, *p* = 0.011).

Regarding PTS (Figure 6), values were comparable between groups in terms of both final measurements and inlier rates, with no significant differences observed.

JL restoration was more consistent with IBRA, with 76.2% (45/59) of patients achieving JL restoration (±2 mm) compared to 31.6% (25/76) in the ILRA group (*p* < 0.001) (Figure 7).

## DISCUSSION

The most important finding of the present study was that IBRA led to superior alignment accuracy and shorter operative time compared to ILRA in lateral UKA. While both IBRA and ILRA systems showed satisfactory results, IBRA demonstrated significantly higher precision, particularly in achieving post‐operative alignment within the target zone and in restoring JL height.

Achieving precise post‐operative HKA alignment is critical for the success of lateral UKA [29], as malalignment can lead to uneven load distribution, joint instability, progression of medial compartment osteoarthritis, and premature implant failure [9, 32]. Our results underscore the superior accuracy of the IBRA system in aligning the mechanical axis, and are consistent with prior literature emphasizing the importance of robotic assistance in enhancing alignment accuracy in lateral UKA [10, 12, 13, 27, 28, 33, 35]. A similar study on medial UKA by Gaggiotti et al. [12] demonstrated that IBRA reduced the rate of radiologic outliers for JL (81.1% vs. 69.5%, *p* = 0.04) and PTS (93.7% vs. 82.7%, *p* = 0.01) when compared to ILRA. However, no significant difference was observed for HKA inliers (77.9% for IBRA vs. 67.5%, *p* = 0.07). The statistical significance of HKA inliers observed in our study could be attributed to the differences in the biomechanics and surgical complexity of lateral UKA compared to medial UKA. Lateral UKA presents unique challenges due to the asymmetrical anatomy and specific kinematics of the lateral compartment, including the screw‐home mechanism, which requires subtle internal rotation of the tibial component to accommodate physiological motion. These results are consistent with the literature showing that conventional instrumentation in lateral UKA is less reliable for achieving accurate alignment. In particular, Batailler et al. [2] demonstrated that robotic assistance significantly improves the reproducibility of implant positioning compared to manual techniques, with fewer radiologic outliers. While such adjustments are difficult to achieve with conventional mechanical instrumentation, robotic assistance offers enhanced preoperative planning and intraoperative control, potentially allowing more accurate replication of native rotational alignment. Furthermore, the volume of lateral UKA procedures is significantly lower compared to medial UKA [26, 30], resulting in reduced familiarity among surgeons and potentially greater variability in radiological outcomes. This is supported by Batailler et al. [2], who reported a higher prevalence of outliers in lateral UKA compared to medial UKA when using ILRA. This variability may have amplified the comparative advantage of IBRA in achieving precise post‐operative alignment in our study. In contrast, the higher volume and standardization of medial UKA procedures in Gaggiotti et al.'s cohort [12] may have minimized the observed differences between IBRA and ILRA systems. While both systems in our study performed well overall, the enhanced preoperative planning facilitated by the IBRA's 3D imaging likely contributed to its superior precision in achieving the target HKA range. While our findings support the precision advantage of robotic assistance, it remains unclear whether this improved alignment translates into better long‐term clinical outcomes, particularly in lateral UKA. Maritan et al. [20] reported similar 5‐year survivorship between RA and conventional lateral UKA, highlighting the need for longer‐term studies to determine whether increased radiographic accuracy leads to improved implant longevity. Nevertheless, an important limitation is the requirement for preoperative CT imaging with the IBRA system. While this enables detailed preoperative planning and may enhance accuracy, it also introduces additional costs, patient irradiation and logistical complexity. These constraints may limit the broader adoption of IBRA in certain centres and must be weighed against the potential benefits in precision.

In our study, the IBRA group showed a significantly smaller change in HKA between preoperative and post‐operative measurements compared to the ILRA group, suggesting better preservation of native limb alignment. While a target zone of 180–185° was used for valgus knees, recent studies increasingly support a more personalized approach—particularly in lateral UKA, where preserving a slight residual valgus (typically 3°–7°) [21] appears to better respect native biomechanics and improve functional outcomes. In particular, van der List et al. [18] reported superior results when a mild valgus was maintained, especially with IBRA systems. These findings challenge the historical emphasis on mechanical neutrality and support interpreting alignment outcomes not only by proximity to a predefined target, but also by the system's ability to avoid overcorrection and maintain anatomical fidelity.

Restoration of JL height is a crucial factor for preserving the native kinematics of the knee, particularly in the lateral compartment [15, 22, 24]. In our analysis, 76.2% of IBRA cases achieved the target JL restoration (±2 mm), compared to only 31.6% of ILRA cases. This significant difference highlights the advantage of IBRA's detailed preoperative planning, which allows for more precise component positioning. JL height restoration has been studied less extensively in lateral UKA than in medial UKA [9, 12, 15, 24, 28]. These findings align with Gaggiotti et al. [12], who reported improved JL height accuracy in medial UKA with IBRA (81.1% vs. 69.5%, *p* = 0.04). The high variability observed in the ILRA group may stem from its reliance on intraoperative bone mapping, which lacks the detailed preoperative planning offered by IBRA's 3D models. Both systems, however, performed better than manual techniques, which often report JL restoration rates below 30% [1, 24].

Accurate restoration of the PTS is essential for maintaining knee stability and preventing implant overloading. Our study found no significant differences in post‐operative PTS between the IBRA and ILRA systems, with both achieving comparable outcomes (mean PTS: 4.2 ± 3.0° for IBRA vs. 3.8 ± 2.4° for ILRA), with most cases falling within the predefined target range of 2° to 8° (66.1% for IBRA vs. 69.7% for ILRA). This target was chosen based on commonly accepted thresholds in the literature [5, 16], though it does not account for each patient's native slope. Rather than aiming systematically for anatomical slope restoration, surgical planning prioritized optimal soft tissue balance, which may have influenced the final PTS achieved. The robotic systems allowed precise control of slope within this range while adapting to intraoperative ligament tension and joint behaviour. These results are consistent with prior studies suggesting that RA systems, regardless of type, are highly effective in achieving precise PTS [10, 28, 34] and significantly improve manual techniques. While the IBRA's preoperative imaging may offer a theoretical advantage in PTS planning, the intraoperative feedback provided by the ILRA system appears to mitigate this difference in practice.

An important secondary finding of our study was the significantly shorter surgical duration associated with the IBRA system (61.6 ± 13.5 min for IBRA vs. 81.9 ± 26.1 min for ILRA). The reduced operative times observed with IBRA systems are likely attributed to their reliance on preoperative imaging for detailed planning, which minimizes the need for extensive intraoperative adjustments. Additionally, the use of an integrated robotic saw in IBRA systems may expedite bone preparation compared to the burr‐based technique required by ILRA systems. Furthermore, the ILRA system was the first robotic platform introduced in our institution, and the initial learning curve may have contributed to longer operative durations during its early adoption phase, despite this effect diminishing over time. This efficiency advantage is particularly beneficial in high‐volume surgical centres, where shorter operative times can improve workflow, enhance patient turnover and potentially reduce overall costs.

Despite its strengths, our study has several limitations that must be acknowledged. First, the retrospective design inherently limits the ability to control for confounding variables. While we attempted to match patient groups as closely as possible, unmeasured factors may still have influenced the results. Second, our analysis focused exclusively on radiographic outcomes, with no assessment of functional or patient‐reported outcomes. Post‐operative radiographs were not acquired under fluoroscopic control, which may have introduced variability in patient positioning, limb rotation, and beam angle. These factors are known to affect the accuracy and reproducibility of coronal (HKA) and sagittal (PTS) measurements, especially when differences between groups are small. Although efforts were made to standardize imaging protocols across all patients, the inherent limitations of conventional radiography could have influenced the precision of our alignment and slope assessments, and must be considered when interpreting the results. To mitigate this limitation, inter‐ and intra‐observer reliability analyses were conducted and demonstrated high reproducibility of the radiographic measurements. Future studies should include long‐term follow‐up and functional assessments to provide a more comprehensive evaluation of these robotic systems. Additionally, our study was conducted at a single centre with a small number of experienced surgeons, which may limit the generalizability of the findings. The learning curve associated with RA systems, particularly IBRA, was not evaluated, and it is possible that less experienced surgeons may achieve different results. Finally, we did not evaluate cost‐effectiveness, which is an important consideration in the adoption of advanced robotic systems.

This study provides new evidence highlighting the superior precision and efficiency of IBRA over ILRA in achieving optimal alignment and JL restoration in lateral UKA, contributing valuable insights to the limited literature on RA techniques for this less common procedure.

## CONCLUSION

Lateral UKA using an IBRA system demonstrated superior accuracy in achieving post‐operative alignment and JL restoration within preoperatively defined target zones, compared to ILRA, along with a significant reduction in operative time. These findings highlight the advantages of IBRA in enhancing the precision and efficiency of lateral UKA, contributing valuable insights towards the optimization of RA knee surgery.

## AUTHOR CONTRIBUTIONS

*Study design, manuscript writing, data collection and statistical analysis*: Clément Favroul. *Study design and manuscript editing*: Cécile Batailler. *Data collection*: Elsayed Ahmed Abdelatif. *Manuscript editing*: Elvire Servien. *Study design, supervision, literature review and manuscript editing*: Sébastien Lustig. All authors read and approved the final manuscript.

## CONFLICT OF INTEREST STATEMENT

Cécile Batailler: Consultant for Stryker, Smith & Nephew. Elvire Servien: Consultant for Corin. Sébastien Lustig: Consultant for Stryker, Smith & Nephew, Heraeus, Depuy Synthes; Institutional research support from Groupe Lepine, Amplitude; Editorial Board for Journal of Bone and Joint Surgery (Am). The remaining authors declare no conflicts of interest.

## ETHICS STATEMENT

All procedures were performed in accordance with the ethical standards of the institutional and/or national research committee, the 1964 Helsinki Declaration and its later amendments, or comparable ethical standards. Data collection and analysis were carried out in accordance with MR004 Reference Methodology from the Commission Nationale de l'Informatique et des Libertés (Ref. 2229975V0) obtained on 6 May 2023. The study was registered and filed on the Health Data Hub website. As per institutional standards, formal patient consent is not required for this type of study.

## Data Availability

Data available on request due to privacy/ethical restrictions.
